# Identification of differentially expressed genes and the role of PDK4 in CD14^+^ monocytes of coronary artery disease

**DOI:** 10.1042/BSR20204124

**Published:** 2021-04-06

**Authors:** Pei Du, Ren Guo, Keqin Gao, Shuang Yang, Baige Yao, Haobo Cui, Ming Zhao, Sujie Jia

**Affiliations:** 1 Department of Pharmacy, The Third Xiangya Hospital, Central South University, Changsha 410013, Hunan, China; 2 Department of Dermatology, The Second Xiangya Hospital, Central South University, Hunan Key Laboratory of Medical Epigenomics, Changsha 410013, Hunan, China; 3 Department of Pharmacy, Weifang People’s Hospital, Weifang 261000, Shandong, China

**Keywords:** coronary artery disease, differentially expressed genes, monocytes

## Abstract

*Background*. Coronary artery disease (CAD) is a chronic inflammatory disease caused by development of atherosclerosis (AS), which is the leading cause of mortality and disability. Our study aimed to identify the differentially expressed genes (DEGs) in CD14^+^ monocytes from CAD patients compared with those from non-CAD controls, which might pave the way to diagnosis and treatment for CAD. *Methods*. The RNA-sequencing (RNA-seq) was performed by BGISEQ-500, followed by analyzing with R package to screening DEGs. Gene Ontology (GO) and Kyoto Encyclopedia of Genes and Genomes (KEGG) pathway analyses were performed by R package. In addition, we validated the results of RNA-seq using real-time quantitative polymerase chain reaction (RT-qPCR). Furthermore, we explored the function of selected ten genes in LDL-treated CD14^+^ monocytes by RT-qPCR. *Results*. a total of 2897 DEGs were identified, including 753 up- and 2144 down-regulated genes in CD14^+^ monocytes from CAD patients. These DEGs were mainly enriched in plasma membrane and cell periphery of cell component, immune system process of biological process, NF-κB signaling pathway, cell adhesion molecules signaling pathway and cytokine–cytokine receptor interaction signaling pathway. In LDL-treated CD14^+^ monocytes, the mRNA expression of pyruvate dehydrogenase kinase 4 (PDK4) was significantly up-regulated. *Conclusion*. In the present study, we suggested that PDK4 might play a role in progression of CAD. The study will provide some pieces of evidence to investigate the role and mechanism of key genes in the pathogenesis of CAD.

## Introduction

Coronary artery disease (CAD) is a heart disease caused by atherosclerotic lesions leading to progressive stenosis of the coronary arteries, resulting in myocardial ischemia, hypoxia, or necrosis. In recent years, CAD has become one of the leading cause of human deaths worldwide [1].

The development of CAD is a long-term chronic inflammatory process. Atherosclerosis (AS) is the pathophysiological basis of CAD. The characteristics of AS include endothelial dysfunction, vascular inflammation, and plaque formation [2,3]. Endothelial dysfunction is the first step in AS, and damaged endothelial cells release large amounts of cytokines and pro-inflammatory mediators, leading to vascular inflammation [4,5]. When the endothelial function of the arterial wall is disordered, lipid accumulation deposits on the intima of the damaged blood vessel and is swallowed by macrophages, thereby forming foam cells and accumulating, eventually leading to plaque formation [6].

Monocytes participate in all aspects of the development of AS and promote the development of atherosclerotic plaque [7]. In the early stage of AS, a large number of blood cells, mainly monocytes and T lymphocytes, adhere to the endothelium of the arterial injury site, which becomes a marker of early atherosclerotic lesions [8,9]. Monocytes adhering to the endothelium migrate to the subendothelial of the endothelium under the action of various cytokines [10], and then differentiate into macrophages or dendritic cells to phagocytose lipids in the subendothelial to form foam cells [7]. Studies have found that the levels of CD14^++^CD16^+^CCR2^+^(Mon2) monocyte are significantly elevated in patients with acute coronary syndrome [11]. Activated monocytes also recruit leukocytes to plaque sites and activate downstream signaling pathways, such as the NF-κB signaling pathway [12], and secrete a variety of biologically active molecules such as enzymes, plasma proteins, and cytokines [13]. Activation of monocytes and T cells in plaques can lead to plaque rupture, leading to acute myocardial infarction or stroke [14]. These results suggest that activated monocytes play a key role in the development of AS and CAD. However, the molecular mechanism of monocyte activation in CAD has not been fully elucidated.

In recent years, the high-throughput sequencing has been used to identify the differentially expressed genes (DEGs) in peripheral blood of CAD patients. For example, Liu et al. [15] found that 12 genes associated with Toll-like receptor signaling pathways are closely related to the occurrence and severity of CAD by transcriptome sequencing of peripheral blood in 21 patients with CAD and 9 healthy volunteers. Arvind et al. [16] found that 190 genes were abnormally expressed in peripheral blood of patients with CAD compared with the negative control by transcriptome sequencing, of which 142 genes were up-regulated and 48 genes were down-regulated, suggesting aberrant gene expression are closely related to the occurrence and development of CAD. Kashyap et al. [17] found 394 DEGs using transcriptome sequencing, and these DEGs were associated with the severity of CAD. A study has shown that 105 DEGs which included 82 up-regulated and 232 down-regulated genes were screened by transcriptome sequencing in CD133^+^ bone marrow-derived progenitor cells from patients with CAD [18]. These results indicated that aberrant gene expression profiling was involved in the immune cells of CAD patients.

In the present study, we performed RNA-sequencing (RNA-seq) to compare the gene expression profiling of CD14^+^ monocytes in CAD patients with controls. DEGs that up-regulated or down-regulated were enriched by gene ontology (GO) functional annotation and Kyoto Encyclopedia of Genes and Genomes (KEGG) pathway analysis. Moreover, we chose 10 DEGs to validate in CD14^+^ monocytes from 18 CAD patients and 18 controls. In addition, we also detected the expression of 4 genes (FOS-like antigen 2 (*FOSL2*), fructose-2,6-biphosphatase 3 (*PFKFB3*), pyruvate dehydrogenase kinase 4 (*PDK4*), and prostaglandin E receptor 4 (*PTGER4*)) among the 10 genes in the CD14^+^ monocytes treated with LDL *in vitro*. The above results will provide some pieces of evidence to investigate the role and mechanism of key genes in the pathogenesis of CAD.

## Materials and methods

### Subjects

RNA-seq was performed in 11 CAD patients who diagnosed by angiography (at least one coronary artery with a stenosis ≥50% of the diameter) and 9 age- and sex-matched angiographically defined non-CAD controls (without coronary artery with a stenosis ≥ 50% of the diameter). Validation of RNA-seq data was performed by real-time quantitative polymerase chain reaction (RT-qPCR) in 18 CAD patients diagnosed by angiography and 18 age- and sex-matched angiographically defined non-CAD controls (without coronary artery with a stenosis ≥ 50% of the diameter). All samples were recruited from the Third Xiangya Hospital. The clinical characteristics of patients and controls were shown in Supplementary Table S1. We excluded the diseases which were autoimmune disease, participants with cardiomyopathy, peripheral vascular disease, malignant disease, thyroid diseases, severe kidney, liver disease, or the condition using statins before blood sampling. The present study was approved by the Ethics Committee of the Third Xiangya Hospital of Central South University, and written informed consent was obtained from all subjects.

### Isolation of CD14^+^ monocyte cells

Peripheral blood mononuclear cells (PBMCs) were isolated via Ficoll-Hypaque density gradient centrifugation (GE Healthcare, Boston, MA, U.S.A.). CD14^+^ monocytes were then isolated from PBMCs by positive selection using magnetic beads (Miltenyi Biotec, Bergisch Gladbach, Germany; the purity was generally greater than 95%) according to the protocol provided by the manufacturer.

### RNA-seq and data analysis

RNA-seq-based transcriptome profiling was performed by Beijing Genomics Institute (Wuhan), using the BGISEQ-500 platform. After extraction, the total RNA was checked by Agilent Bioanalyzer 2100 (Agilent Technologies, Inc., Santa Clara, CA). The mRNA was enriched by oligo (dT) magnetic beads to enrich mRNA and deplete rRNA and was fragmented into short fragments using fragment buffer. With the mRNA fragments as templates, the first-strand cDNA was synthesized by random N6 primer. Then the second strand was synthesized with the first-strand cDNA as templates. The synthetic double-stranded cDNA ends were flattened and phosphorylated at the 5′ end and formed a sticky jointed with a bubble-like convex at the 3′ end. The connected products were amplified by polymerase chain reaction to produce the sequencing library. The sequencing was performed by BGISEQ-500.

Subsequent filtering was performed using the SOAPnuke software. After filtering, the sequencing data were mapped to the human reference genome hg38 using HISAT and Bowtie2 software. Accurate transcript quantification and normalization were performed using RSEM software package. The DEGs were identified by R package.

The annotated DEGs were classified according to biological process, cellular component, and molecular function using Phyper function of R software package. The annotated DEGs were enriched in different pathways according to cellular processes, environmental information processing, genetic information processing, human diseases, metabolism and organismal systems using Phyper function of R software package.

### Cell culture and treatment

CD14^+^ monocytes were cultured in RPMI 1640 medium (Gibco, New York, U.S.A.) with 10% fetal bovine serum (FBS, Capricorn Scientific, U.S.A.). The cultures were incubated at 37°C in a humidified atmosphere, which contained 5% CO_2_. The CD14^+^ monocytes were seeded at a density of 5 × 10^5^ cells/mL in a 12-well plate, and LDL (Yiyuan Biotechnologies, Guangzhou, China) was added at a final concentration of 100 mg/L for 24 h.

### RNA isolation and RT-qPCR

Total RNA was isolated from CD14^+^ monocytes using TRIzol reagent (Invitrogen, Carlsbad, CA, U.S.A.) according to the manufacturer’s instructions. RT-qPCR was performed using a Light Cycler 96 (Roche, Basel, Switzerland), and the mRNA levels were quantified using a SYBR Prime Script RT-qPCR kit (Takara, Dalian, China). β-actin was also amplified and used as a loading control. The relative expression of mRNA was calculated using 2^−Δ*C*_t_^ (Δ*C*_t_ = *C*t_target gene_ − *C*t_β-actin_) method in CAD patients compared with controls and the fold change of mRNA expression level was calculated using 2^−ΔΔ*C*_t_^ method (ΔΔ*C*_t_ = Δ*C*t_experiment group_ − Δ*C*t_control group_) in experiment group compared with control group. All data were normalized to internal control β-actin. The sequences of the primers are shown in Supplementary Table S1.

### Statistical analysis

All the data were analyzed by SPSS 19.0 software (SPSS, Inc., Chicago, IL). The measurement data were expressed as mean ± standard deviation (SD), the normal distribution of the data was detected by the D’Agostino–Pearson omnibus normality test, and comparison between two groups was done with non-parametric tests (data from LDL treatment was compared by the Wilcoxon’s signed-rank test, data from CAD patients and controls was compared by the Mann–Whitney U test). A *P*-value (two-sided) <0.05 was considered statistically significant.

## Results

### Comparison of gene expression profiles in CD14^+^ monocytes between CAD patients and non-CAD controls

**Figure 1:**
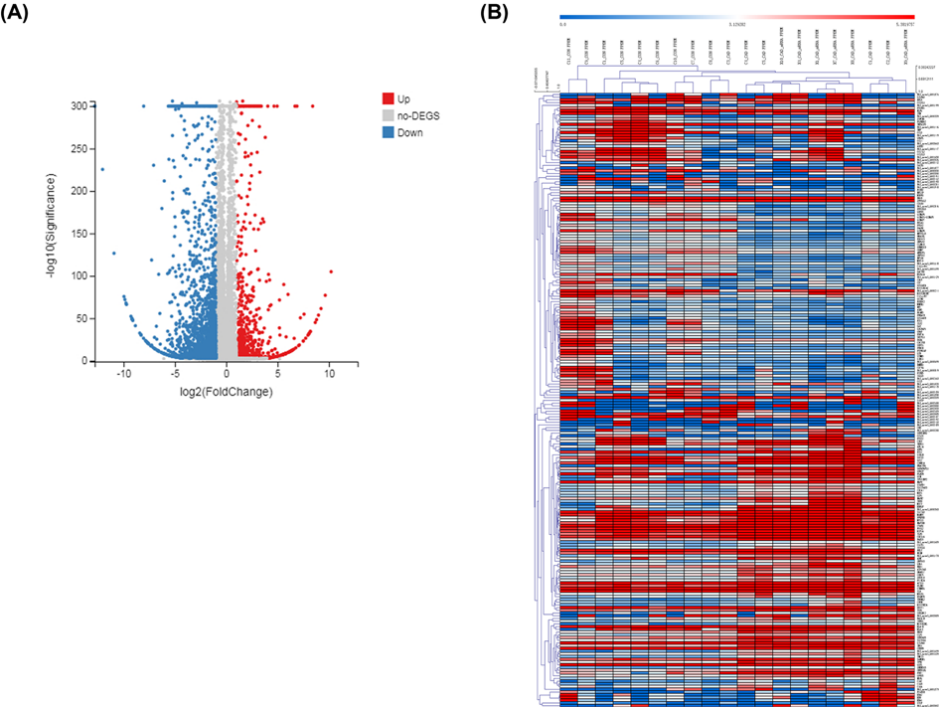
DEGs in CAD patients compared with non-CAD controls (**A**) Volcano plot showed the −log_10_ (*P*-value) and log_2_ (fold change) for all expressed genes. The differentially up-regulated genes were indicated by red dots, and the differentially down-regulated genes were indicated by blue dots. (**B**) Heatmaps of 225 DEGs were filtered by *P*<0.05, FPKM > 2, and log2(fold change) > 3. Normalized gene expression is represented across each row, where red indicates higher values and blue indicates lower values.

To identify whether there are differences in gene expression profiles in CD14^+^ monocytes between CAD patients and controls, we performed RNA-seq to detect transcriptome expression profiles of 11 CAD patients and 9 controls. The clinical characteristics of all subjects are shown in Supplementary Table S2. Compared with controls, a total of 2897 DEGs were identified, among which 753 genes were found to be up-regulated and 2144 genes were found to be down-regulated in CD14^+^ monocytes from CAD patients (*P*<0.05 and fold change > 2.0) (Supplementary Table S3 and Figure 1A). After filtering, 225 genes were differentially expressed with *P*<0.05, Fragments Per Kilobase of exon model per Million mapped fragments (FPKM) > 2 and log2(fold change) > 3. Hierarchical clustering of the data was depicted by heat map (Figure 1B).

### GO analysis of DEGs

**Figure 2:**
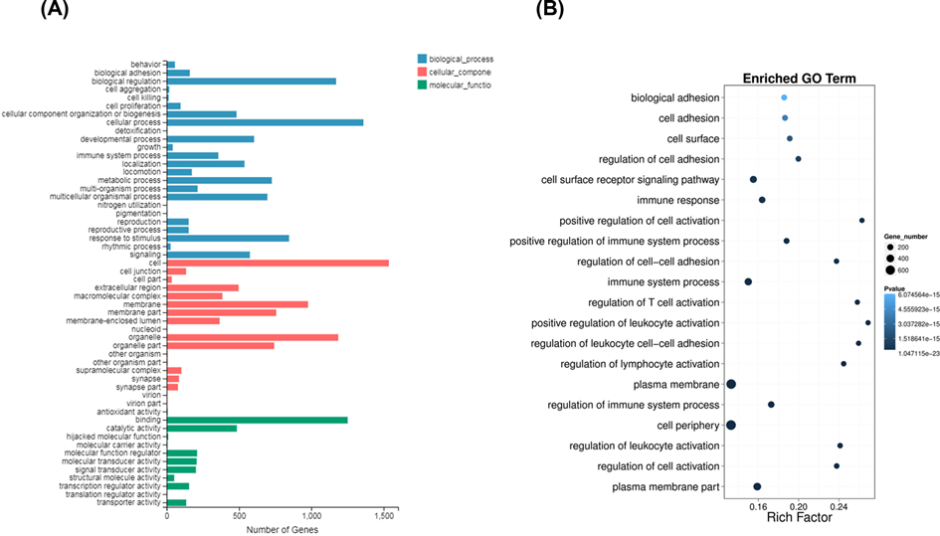
GO analysis of DEGs (**A**) The GO slim summary for DEGs was enriched in biological process, cellular component, and molecular function. (**B**) The bubble map showed top 20 significantly enriched terms.

The GO slim summary for all DEGs containing 54 categories was shown in Figure 2A. Among which, most DEGs (1360/1571) were enriched in cellular process and 1171 genes were enriched in biological regulation for biological process, followed by response to stimulus (846/1571). Most DEGs were associated with organelle (1186/1731) and membrane (977/1731) for cellular component. The molecular function of DEGs was enriched in binding (1251/1505) and catalytic activity (485/1505). The top 20 significantly enriched terms in biological process were shown in Figure 2B. The DEGs were significantly enriched in regulation of leukocyte activation, regulation of immune system process, regulation of cell adhesion and cell surface receptor signaling pathway. Some DEGs were significantly enriched in lymphocyte activation and T-cell activation, which suggested the molecular basis of activation and migration of CD14^+^ monocytes in CAD patients. The significant top 20 enrichments of GO terms are shown in Supplementary Table S4.

### KEGG analysis of DEGs

**Figure 3:**
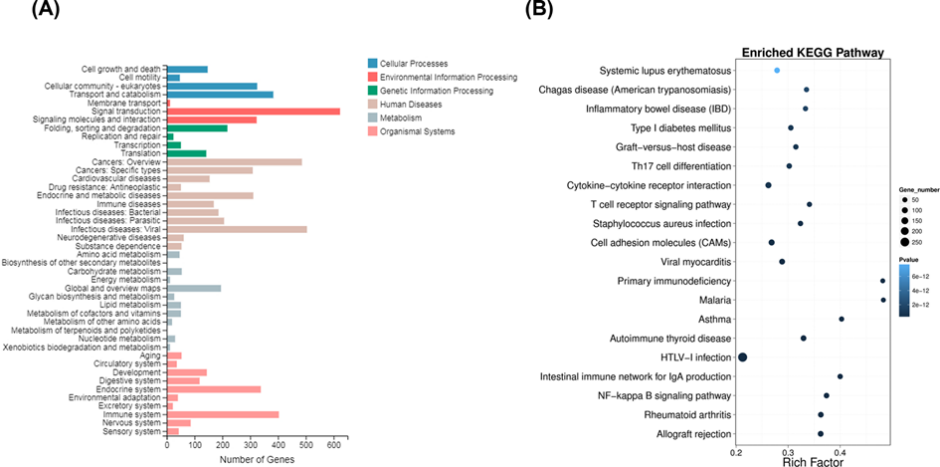
KEGG analysis of DEGs (**A**) The enriched KEGG pathways of all DEGs were listed with 44 categories. (**B**) The bubble map showed top 20 significantly enriched KEGG pathways.

As shown in Figure 3A, the enriched KEGG pathways for all DEGs contained 44 categories. Among which, 383 genes were enriched in transport and catabolism for cellular processes; 623 genes were enriched in signal transduction for environment information processing; 504 genes were enriched in infectious diseases: viral and 486 genes were enriched in cancers: overview for human diseases; 403 genes were enriched in immune system and 338 genes were enriched in endocrine system for organismal systems. The top 20 significant KEGG pathways are shown in Figure 3B. The DEGs were significantly enriched in NF-κB signaling pathway, cell adhesion molecules signaling pathway, and cytokine–cytokine receptor interaction signaling pathway, which suggested the differential expression of genes in these pathways may contribute to the aberrant activation and function of CD14^+^ monocytes in CAD patients were closely related to CAD process. The significant top 20 enrichments of KEGG pathways are shown in Supplementary Table S5.

### Validation of DEGs in RNA-seq

**Figure 4:**
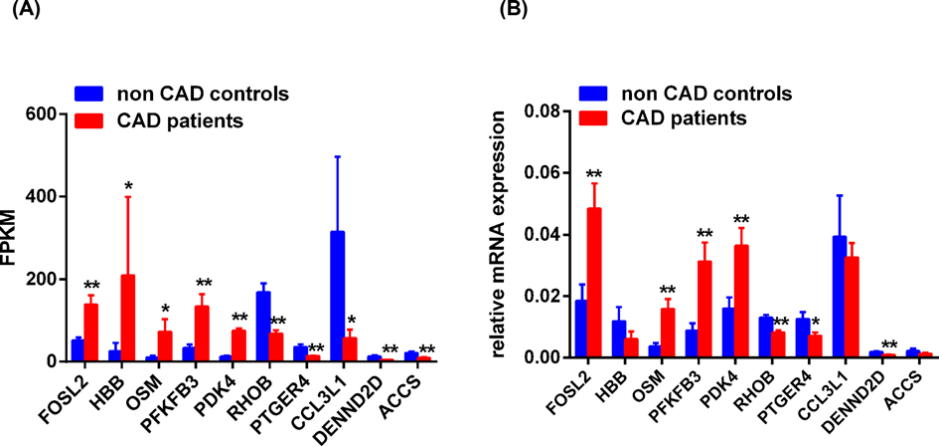
Validation of DEGs in RNA-seq (**A**) The normalized expressed values of selected ten DEGs were depicted in sequencing data. (**B**) The relative expression of selected ten DEGs was used for validating of RNA-seq. *P<0.05 relative to control, **P<0.01 relative to control.

To assess the reliability of data of RNA-seq, five genes (*FOSL2, HBB, OSM, PFKFB3, PDK4*) were selected in up-regulated genes and five genes (*RHOB, PTGER4, CCL3L1, DENND2D, ACCS*) were selected in down-regulated genes for detection in 18 CAD patients and 18 controls by RT-qPCR according to expression value and significance. In sequencing data, the genes *FOSL2, HBB, OSM, PFKFB3*, and *PDK4* were up-regulated and the genes *RHOB, PTGER4, CCL3L, DENND2D*, and *ACCS* were down-regulated in CD14^+^ monocytes from CAD patients compared with controls (Figure 4A). The mRNA expression of *FOSL2, OSM, PFKFB3*, and *PDK4* were significantly up-regulated and the mRNA expression of *RHOB, PTGER4*, and *DENND2D* were significantly down-regulated using RT-qPCR (Figure 4B). However, there were no significant changes of the genes *HBB, CCL3L*, and *ACCS* between the two groups (Figure 4B). These results suggested that sequencing data were basically consistent with the results detected by RT-qPCR.

### LDL-induced aberrant expressed gene *PDK4* was associated with CAD

**Figure 5:**
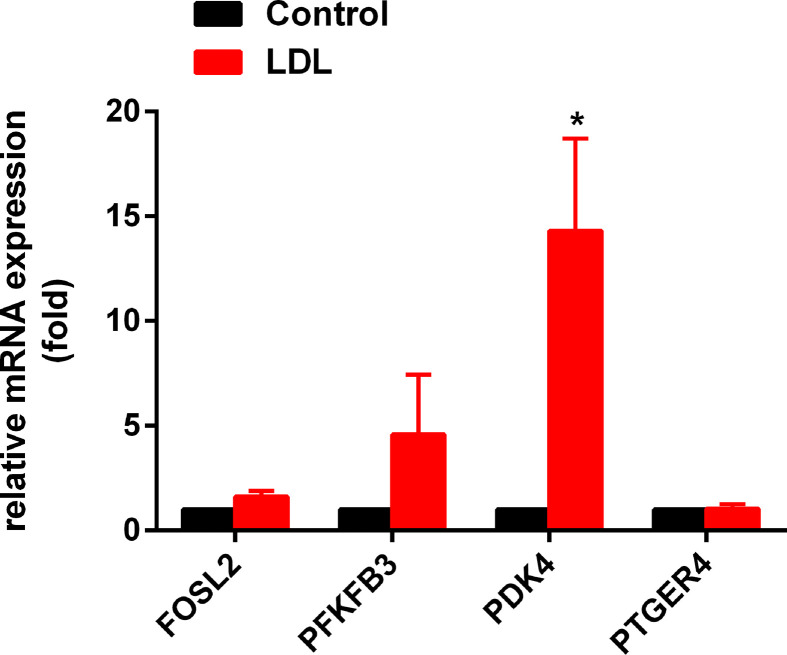
LDL-induced aberrant expressed gene PDK4 was associated with CAD The mRNA expression of FOSL2, PFKFB3, PDK4, and PTGER4 in LDL-treated CD14^+^ monocytes was detected by RT-qPCR (*n*=6). *P<0.05 relative to control.

Among the seven DEGs, four genes including *FOSL2, PFKFB3, PDK4*, and *PTGER* may be associated with the function of CD14^+^ monocytes according to the previous publications.[19–23] Our previous study demonstrated that LDL treatment induced the aberrant activation of CD14^+^ monocytes [24]. Therefore, in this study, we detected the expression changes of four genes including *FOSL2, PFKFB3, PDK4*, and *PTGER4* in CD14^+^ monocytes with LDL treatment to identify the association of them with LDL-induced CD14^+^ monocytes activation. Compare with controls, the mRNA expression of PDK4 was significantly up-regulated in LDL-treated CD14^+^ monocytes (Figure 5). The results suggested that PDK4 may play an important role in abnormal activation of CD14^+^ monocytes in CAD patients.

## Discussion

In the present study, we used RNA-seq to compare the gene expression of CD14^+^ monocytes in CAD with that in controls. The aberrant expression of many genes was identified in CD14^+^ monocytes, which may be associated with the pathogenesis of CAD. These DEGs were enriched by GO functional annotation and KEGG pathway. The DEGs were enriched in plasma membrane and cell periphery of cell component and immune system process of biological process. KEGG pathway analysis found that DEGs were significantly enriched in NF-κB signaling pathway, cell adhesion molecules signaling pathway, and cytokine–cytokine receptor interaction signaling pathway, which were closely related to CAD process. In LDL-treated CD14^+^ monocytes, the mRNA expression of PDK4 was significantly up-regulated, which in accordance with that in CD14^+^ monocytes of CAD patients compared with controls.

GO functional annotation enrichment analysis of DEGs provided several known and novel molecular mechanisms in development of CAD. Our results showed that most genes were enriched in regulation of leukocytic activation, such as tumor necrosis factor superfamily member 9 (TNFSF9), which is up-regulated in CD14^+^ monocytes from CAD patients and may promote differentiation of monocyte-derived dendritic cells [25], mitogen-activated protein kinase kinase kinases 8 (MAP3K8), which is up-regulated in CD14^+^ monocytes from CAD patients and involved in the enhancement of C–C chemokine receptor type 2 (CCR2) expression and abilities of migration and adhesion on Ly6C^high^CD11c^low^ monocytes of ApoE^−/−^ mice fed a high-fat diet [26]. Monocytes play an important role in the development and progress of CAD. In the early stage, monocytes were activated by micro-environment of blood and cytokines released by endothelial cells, subsequently transmigrating to the subendothelial tissue and swallowing lipid to differentiate to foam cells [27]. Studies found that leukocytic activation which included neutrophils, monocytes, and lymphocytes was connected with the severity of CAD [28]. T cell is an important part of early ‘fatty-streak’ lesion, accelerating the chronic inflammatory response in progress of lesion formation [29]. When fibroatheromatous plaques are covered by a fibrous cap, the ‘shoulder’ regions are heavily infiltrated by T cells, which produce enzymes and proinflammatory mediators, contributing to adventitial inflammation of advanced plaques [30]. It is reported that the densities of CD20^+^ B lymphocytes in the plaque perivascular adipose tissue were greater in the presence of larger luminal obstruction by atherosclerotic plaques [31]. Furthermore, CD20^+^ B lymphocytes secreted cytokines, which increased the vulnerability of the atherosclerotic plaques by the recruitment of macrophages and increased the necrotic core in advanced lesions [32]. These results demonstrated that leukocytic activation participated in development of CAD.

KEGG pathway analysis revealed that the DEGs were enriched in NF-κB signaling pathway, cell adhesion molecules signaling pathway, and cytokine–cytokine receptor interaction signaling pathway. DEGs enriched in NF-κB signaling pathway include the up-regulated genes like CXC chemokine CXCL12, vascular cell-adhesion molecule 1 (VCAM1), urokinase-type plasminogen activator (PLAU), interleukin-1 receptor 1 (IL1R1) and down-regulated genes, such as tumor necrosis factor α-induced protein 3 (TNFAIP3), a negative regulator of TLR signaling pathways [33], and tumor necrosis factor receptor-associated factor 5 (TRAF5), suppressing NF-κB signaling pathway [34]. NF-κB is a transcription factor that regulates the expression of key genes in a variety of immune processes. Functional NF-κB is a complex composed of at least one Rel protein (RelA, RelB, or RelC) and p50 or p52 [35,36]. NF-κB regulated the expression of many AS-associated pro-inflammatory genes, and NF-κB signaling pathway were thought to be key to pro-AS due to their important role in vascular inflammation, vascular smooth muscle cell proliferation or foam cell formation [12,37]. Downstream of NF-κB signaling pathway was release of a variety of cytokines and chemokines which amplified inflammatory response to accelerate the progress of CAD [38–42]. The aberrant activation of NF-κB signaling pathway may promote the migration and adhesion of monocytes in vascular endothelium, accelerating the progress of AS. These DEGs enriched in cell adhesion molecules include up-regulated genes, like cell adhesion molecule 3 (CADM3), E-selectin (SELE), and Claudin 5 (CLDN5), and down-regulated genes, such as neuronal guidance protein neogenin 1 (NEO1), which is suppressing in CD14^+^ monocytes from CAD patients and its limited expression could induce the polarization of monocytes [43]. These results reflected that the expression changes of genes in some important pathways may contribute to the aberrant activation and functions of monocytes in CAD patients.

According to expression value and significance, we identified 50 up-regulated genes and 50 down-regulated genes in CAD patients based on the data of RNA-seq (Supplementary Table S6). Furthermore, we selected five up-regulated genes and five down-regulated genes to validate gene expression by RT-qPCR according to the functions of these genes reported by previous publications. FOSL2, is also known as FRA-2, is a component of AP-1 transcription factor family [44]. FOSL2 acts as an important part of cancer [45,46], photoperiodic regulation [47] and T cell differentiation [48]. FOSL2 also regulates the TGF-β signaling pathway, thereby affecting the migration of colon cancer cells [19]. Mutation of HBB, the encoding gene of atypical β globin, is associated with sickle cell anemia, and expression of β-globin promotes cell survival in cancer [49,50]. Oncostatin M (OSM) is a member of the interleukin-6 (IL-6) cytokine family produced by inflammatory cells, which has been reported to induce angiogenesis by up-regulating vascular endothelial growth factor (VEGF) and basic fibroblast growth factor (bFGF) and involve in remodelling of various tissues [51]. PFKFB3 is an enzyme which regulates glycolysis. Studies have shown the role of PFKFB3 in regulation of glycolysis, proliferation, migration, and angiogenesis of cancer cells [52–54]. Interestingly, studies have shown that PFKFB3 as a glycolysis-regulatory enzyme plays an important role in inflammation of macrophages [20], which suggest PFKFB3 is associated with function of pro-inflammation and migration in CD14^+^ monocytes. PDK4 is a member of pyruvate dehydrogenase kinases which are responsible for the switch from mitochondrial oxidation to cytoplasmic glycolysis and phosphorylating pyruvate dehydrogenase [21]. GTPase RhoB (RHOB) is an important regulator of cytoskeletal organization and vesicle and membrane receptor trafficking [55,56]. The prostaglandin receptor EP4 (PTGER4) is a receptor of PGE2, regulating the action of PGE2 in inflammatory response [57]. Studies have shown that mRNA expression of PTGER4 was up-regulated in THP-1 cells with LPS treatment, following inflammatory activation [22]. The MIP-1α (encoded by CCL3L1) plays an important role in inflammatory responses and regulating of macrophage function [23]. Differentially expressed in normal and neoplastic cells domain containing 2D (DENND2D) is a tumor suppressor gene, regulating Rab GTPases and involved in cancer cell proliferation [58]. *ACCS* gene encodes the 1-aminocyclopropane-1-carboxylate synthase and has been studied more in plants. These results suggested that four genes including *FOSL2, PFKFB3, PDK4*, and *PTGER4* might be involved in function of migration and pro-inflammation in CD14^+^ monocytes. So we detected the mRNA expression of four genes in LDL-treated CD14^+^ monocytes. Abnormal glucose metabolism is involved in progression of CAD which is a chronic inflammatory disease. Monocytes, components of the innate immune system, play an essential role in the initiation and development of AS. As is reported, in monocytes of patients with CAD, overutilization of glucose promotes excessive and prolonged production of the cytokines IL-6 and IL-1β, driving systemic and tissue inflammation [59]. PDK4 is a pyruvate dehydrogenase kinase, regulating glycolysis and phosphorylating pyruvate dehydrogenase [21]. Studies have revealed the up-regulation of PDK4 induced by inflammation in C2C12 cells [60]. And the expression of PDK4 was up-regulated in LDL-treated CD14^+^ monocytes, suggesting that PDK4 might be involved in inducement of aberrant glycolysis in LDL-treated CD14^+^ monocytes and associated with abnormal activation of CD14^+^ monocytes in CAD patients.

## Conclusions

In summary, we identified 2897 DEGs in CD14^+^ monocytes from CAD patients and further studied these genes through GO functional annotation and KEGG pathway analysis. Moreover, we chose 10 DEGs to validate in CD14^+^ monocytes from 18 CAD patients and 18 controls. In addition, we found that PDK4 was up-regulated in the CD14^+^ monocytes treated with LDL *in vitro*. These findings will provide important clues for investigation of the molecular mechanism of CAD. However, we will study the roles of the DEGs in the activation and migration of CD14^+^ monocytes and the pathogenesis of CAD in the future, as well as the upstream mechanism resulting in the changes of gene expression.

## Supplementary Material

Supplementary Tables S1-S6

## Data Availability

All data are available from the corresponding author on reasonable request.

## Abbreviations

AS: atherosclerosis
bFGF: basic fibroblast growth factor
CAD: coronary artery disease
CADM3: cell adhesion molecule 3
CCR2: C-C chemokine receptor type 2
CLDN5: Claudin 5
DEG: differentially expressed gene
DENND2D: differentially expressed in normal and neoplastic cells domain containing 2D
FOSL2: FOS-like antigen 2
GO: gene ontology
IL1R1: interleukin-1 receptor 1
IL-6: interleukin-6
KEGG: Kyoto Encyclopedia of Genes and Genomes
LDL: low density lipoprotein
MAP3K8: mitogen-activated protein kinase kinase kinases 8
NEO1: neuronal guidance protein neogenin 1
OSM: Oncostatin M
PBMC: peripheral blood mononuclear cell
PDK4: pyruvate dehydrogenase kinase 4
PFKFB3: fructose-2,6-biphosphatase 3
PGE2: prostaglandin E2
PLAU: urokinase-type plasminogen activator
PTGER4: prostaglandin E receptor 4
RHOB: GTPase RhoB
RNA-seq: RNA-sequencing
RT-qPCR: real-time quantitative polymerase chain reaction
SELE: E-selectin
TNFAIP3: tumor necrosis factor alpha-induced protein 3
TNFSF9: tumour necrosis factor superfamily member 9
TRAF5: tumor necrosis factor receptor-associated factor 5
VCAM1: vascular cell-adhesion molecule 1
VEGF: vascular endothelial growth factor
