# Supplementary material for: Identification of differentially expressed genes and the role of PDK4 in CD14+ monocytes of coronary artery disease
Source: Biosci Rep. 2021 Apr 6;41(4):BSR20204124. doi: 10.1042/BSR20204124 (PMC8024870; doi:10.1042/BSR20204124)
Supplement: Supplementary Tables S1-S6 [file BSR-2020-4124_supp.zip › BSR-2020-4124_suppST1.docx]

**Supplementary table 1. Primer sequences**

| Gene | Primer | Sequence (5’–3’) |
| --- | --- | --- |
| FOSL2 | Forward | GCGTGATCAAGACCATTGGC |
|  | Reverse | CTTCTCCTCCTCCAGCTCCT |
| HBB | Forward | GGTGCCTTTAGTGATGGCCT |
|  | Reverse | GTTCTCAGGATCCACGTGCA |
| OSM | Forward | GCTTCCTGCAGACCCTCAAT |
|  | Reverse | ATGTTGTTCCTGAGCCCGAG |
| PFKFB3 | Forward | GAGAACGAGCACAACCTCCA |
|  | Reverse | GGTCCTTCAGGTTCTGCTCC |
| β-actin | Forward | GCACCACACCTTCTACAATGAGC |
|  | Reverse | GGATAGCACAGCCTGGATAGCAAC |
| PDK4 | Forward | CCTGTGAGACTCGCCAACAT |
|  | Reverse | GCTTTCTGGTCATCTGGGCT |
| RHOB | Forward | CCGAGGTGAAGCACTTCTGT |
|  | Reverse | GCAGAGCACTCGAGGTAGTC |
| PTGER4 | Forward | GAACCCCATCCTAGACCCCT |
|  | Reverse | ACTGTCTGAGCAGTGCTGTC |
| CCL3L | Forward | AGCTACACCTCCCGACAGAT |
|  | Reverse | CACTCAGCTCCAGGTCACTG |
| DENND2D | Forward | GTTCAGAGGATGATTACGAGCC |
|  | Reverse | GCCCACTCATTCCCATCTGG |
| ACCS | Forward | GAAGGAGAATGCTCCAGAAAACT |
|  | Reverse | CCATGTGGTAGGTCCTGTAGC |
