# Supplementary material for: Identification of differentially expressed genes and the role of PDK4 in CD14+ monocytes of coronary artery disease
Source: Biosci Rep. 2021 Apr 6;41(4):BSR20204124. doi: 10.1042/BSR20204124 (PMC8024870; doi:10.1042/BSR20204124)
Supplement: Supplementary Tables S1-S6 [file BSR-2020-4124_supp.zip › BSR-2020-4124_suppST2.docx]

**Supplementary table 2. Clinical characteristics of CAD patients and controls in RNAseq and validation**

| Characteristics | RNA-seq | | validation | |
| --- | --- | --- | --- | --- |
|  | CAD (n=11)  (Mean ± SD) | Control (n=9)  (Mean ± SD) | CAD (n=18)  (Mean ± SD) | Control (n=18)  (Mean ± SD) |
| Age(yrs) | 60.6 ± 10.8 | 64.2 ± 9.3 | 63.50 ± 10.00 | 62.78 ± 7.84 |
| Gender(M/F) | (5/6) | (6/3) | (6/12) | (8/10) |
| Hypertension(n, %) | 6 (54.6) | 5 (55.6) | 9 (50.00) | 10 (55.56) |
| diabetes(n, %) | 0 | 2 (22.2) | 2 (11.11) | 5 (27.78) |
| SBP(mmHg) | 132.94 ± 17.32 | 133.15 ± 21.31 | 125.94± 33.49 | 135.26 ± 20.25 |
| DBP(mmHg) | 76.59 ± 12.24 | 81.67 ± 11.93 | 78.24 ± 13.74 | 81.65 ± 9.24 |
| HR(bpm) | 78.90 ± 8.46 | 72.26 ± 8.54 | 73.50 ± 15.73 | 70.95 ± 6.80 |
| BNP(pg/mL) | 1225.98 ± 184.39 | 427.28 ± 567.91 | 1144.49 ± 1805.87 | 469.54 ± 97.13 |
| MONO(10^9/L) | 0.48 ± 0.27 | 0.40 ± 0.17 | 0.41 ± 0.13 | 0.37 ± 0.13 |
| TC(mmol/L) | 4.40 ± 0.80 | 4.67 ± 0.93 | 4.37 ± 1.02 | 4.44 ± 0.93 |
| TG(mmol/L) | 1.46 ± 0.50 | 1.40 ± 0.46 | 1.52 ± 0.82 | 1.45 ± 0.58 |
| HDL-C(mmol/L) | 1.16 ± 0.32 | 1.09 ± 0.14 | 1.35 ± 0.25 | 1.10 ± 0.20 |
| LDL-C(mmol/L) | 2.42 ± 0.59 | 2.65 ± 0.71 | 2.47 ± 0.78 | 2.45 ± 0.66 |
| FBS(mmol/L) | 5.06 ± 0.69 | 5.34 ± 0.80 | 5.36 ± 0.59 | 5.30 ± 0.93 |
| ALT(U/L) | 31.81 ± 26.37 | 27.11 ± 17.59 | 28.76 ± 14.68 | 26.28 ± 13.91 |
| AST(U/L) | 21.79 ± 8.73 | 22.22 ± 6.50 | 29.19 ± 12.60 | 23.22 ± 7.10 |
| TBIL(umol/L) | 13.44 ± 5.42 | 14.70 ± 3.27 | 13.69 ± 2.96 | 14.05 ± 5.30 |
| CB(umol/L) | 3.92 ± 2.70 | 3.38 ± 0.76 | 4.57 ± 1.59 | 3.77 ± 1.67 |
| TP(g/L) | 64.51± 4.31 | 64.13 ± 6.70 | 64.74 ± 4.54 | 64.11 ± 5.23 |
| ALB(g/L) | 39.50 ± 1.51 | 41.94 ± 5.36 | 39.15± 2.72 | 40.13 ± 4.24 |
| GLO(g/L) | 28.66 ± 13.94 | 22.19 ± 4.22 | 25.85 ± 2.96 | 23.98 ± 4.17 |
| A/G | 5.18 ± 11.39 | 1.96 ± 0.39 | 1.89 ± 0.55 | 1.74 ± 0.39 |
| UREA(mmol/L) | 4.63 ± 1.20 | 4.76 ± 1.30 | 5.14 ± 1.14 | 4.73 ± 1.09 |
| CRE(umol/L) | 75.36 ± 17.63 | 85.22 ± 17.92 | 63.17 ± 11.52* | 76.33 ± 19.57 |
| UA(umol/L) | 364.27 ± 91.88 | 275.22 ± 70.23 | 292.41 ± 52.03 | 288.22 ± 62.71 |

Continuous variables are expressed as means±SDs.

SBP, indicates systolic blood pressure; DBP, diastolic blood pressure; HR, heart rate; BNP, brain natriuretic peptide; MONO, monocyte count; TC, total cholesterol; TG, Triglyceride; HDL-C, high-density lipoprotein cholesterol; LDL-C, low-density lipoprotein cholesterol; FBG, Fasting blood glucose; ALT, alanine aminotransferase; AST, Aspartate aminotransferase; TBIL, total bilirubin; CB, conjugated bilirubin; TP, total protein; ALB, albumin; GLO, globulin; A/G, albumin/globulin; UREA, Serum urea; CRE, creatinine; UA, Serum uric acid.
