# Supplementary material for: Identification of differentially expressed genes and the role of PDK4 in CD14+ monocytes of coronary artery disease
Source: Biosci Rep. 2021 Apr 6;41(4):BSR20204124. doi: 10.1042/BSR20204124 (PMC8024870; doi:10.1042/BSR20204124)
Supplement: Supplementary Tables S1-S6 [file BSR-2020-4124_supp.zip › BSR-2020-4124_suppST4.docx]

**Supplementary table 4. Significant top 20 enrichments of GO terms of differentially expressed genes.**

| GO Term ID | GO Term | Term Candidate Gene Num | Total Candidate Gene Num | Term Gene Num | Total Gene Num | Rich Ratio | P value | Q value |
| --- | --- | --- | --- | --- | --- | --- | --- | --- |
| GO:0044459 | plasma membrane part | 401 | 1731 | 2523 | 17247 | 0.158937772 | 1.05E-23 | 1.05E-19 |
| GO:0050865 | regulation of cell activation | 121 | 1571 | 509 | 15563 | 0.237721022 | 6.77E-20 | 3.39E-16 |
| GO:0002694 | regulation of leukocyte activation | 116 | 1571 | 481 | 15563 | 0.241164241 | 1.23E-19 | 4.11E-16 |
| GO:0071944 | cell periphery | 655 | 1731 | 4929 | 17247 | 0.132886995 | 1.05E-18 | 2.63E-15 |
| GO:0002682 | regulation of immune system process | 245 | 1571 | 1418 | 15563 | 0.172778561 | 1.65E-18 | 3.31E-15 |
| GO:0005886 | plasma membrane | 641 | 1731 | 4817 | 17247 | 0.133070376 | 2.47E-18 | 4.13E-15 |
| GO:0051249 | regulation of lymphocyte activation | 104 | 1571 | 425 | 15563 | 0.244705882 | 3.44E-18 | 4.93E-15 |
| GO:1903037 | regulation of leukocyte cell-cell adhesion | 89 | 1571 | 343 | 15563 | 0.259475219 | 1.98E-17 | 2.48E-14 |
| GO:0002696 | positive regulation of leukocyte activation | 82 | 1571 | 305 | 15563 | 0.268852459 | 3.90E-17 | 4.34E-14 |
| GO:0050863 | regulation of T cell activation | 86 | 1571 | 333 | 15563 | 0.258258258 | 9.53E-17 | 9.54E-14 |
| GO:0002376 | immune system process | 357 | 1571 | 2380 | 15563 | 0.15 | 1.34E-16 | 1.13E-13 |
| GO:0022407 | regulation of cell-cell adhesion | 100 | 1571 | 421 | 15563 | 0.237529691 | 1.36E-16 | 1.13E-13 |
| GO:0002684 | positive regulation of immune system process | 172 | 1571 | 915 | 15563 | 0.187978142 | 1.48E-16 | 1.14E-13 |
| GO:0050867 | positive regulation of cell activation | 82 | 1571 | 312 | 15563 | 0.262820513 | 1.67E-16 | 1.19E-13 |
| GO:0006955 | immune response | 254 | 1571 | 1551 | 15563 | 0.163765313 | 3.69E-16 | 2.47E-13 |
| GO:0007166 | cell surface receptor signaling pathway | 306 | 1571 | 1974 | 15563 | 0.155015198 | 4.61E-16 | 2.88E-13 |
| GO:0030155 | regulation of cell adhesion | 139 | 1571 | 696 | 15563 | 0.199712644 | 1.06E-15 | 6.24E-13 |
| GO:0009986 | cell surface | 151 | 1731 | 790 | 17247 | 0.191139241 | 2.19E-15 | 1.22E-12 |
| GO:0007155 | cell adhesion | 160 | 1571 | 858 | 15563 | 0.186480186 | 3.97E-15 | 2.10E-12 |
| GO:0022610 | biological adhesion | 160 | 1571 | 862 | 15563 | 0.185614849 | 6.07E-15 | 3.04E-12 |
