# Supplementary material for: Identification of differentially expressed genes and the role of PDK4 in CD14+ monocytes of coronary artery disease
Source: Biosci Rep. 2021 Apr 6;41(4):BSR20204124. doi: 10.1042/BSR20204124 (PMC8024870; doi:10.1042/BSR20204124)
Supplement: Supplementary Tables S1-S6 [file BSR-2020-4124_supp.zip › BSR-2020-4124_suppST5.docx]

**Supplementary table 5. Significant top 20 enrichments of KEGG pathways of differentially expressed genes.**

| Pathway ID | Pathway Name | Term Candidate Gene Num | Total Candidate Gene Num | Term Gene Num | Total Gene Num | Rich Ratio | P value | Q value |
| --- | --- | --- | --- | --- | --- | --- | --- | --- |
| ko05330 | Allograft rejection | 87 | 1801 | 240 | 13934 | 0.3625 | 1.48E-20 | 4.83E-18 |
| ko05323 | Rheumatoid arthritis | 82 | 1801 | 226 | 13934 | 0.362831858 | 1.79E-19 | 2.92E-17 |
| ko04064 | NF-kappa B signaling pathway | 74 | 1801 | 198 | 13934 | 0.373737374 | 1.54E-18 | 1.49E-16 |
| ko04672 | Intestinal immune network for IgA production | 66 | 1801 | 165 | 13934 | 0.4 | 1.83E-18 | 1.49E-16 |
| ko05166 | HTLV-I infection | 269 | 1801 | 1266 | 13934 | 0.212480253 | 3.03E-18 | 1.98E-16 |
| ko05320 | Autoimmune thyroid disease | 86 | 1801 | 261 | 13934 | 0.329501916 | 2.49E-17 | 1.36E-15 |
| ko05310 | Asthma | 58 | 1801 | 144 | 13934 | 0.402777778 | 1.49E-16 | 6.96E-15 |
| ko05144 | Malaria | 43 | 1801 | 89 | 13934 | 0.483146067 | 4.57E-16 | 1.87E-14 |
| ko05340 | Primary immunodeficiency | 40 | 1801 | 83 | 13934 | 0.481927711 | 5.39E-15 | 1.96E-13 |
| ko05416 | Viral myocarditis | 92 | 1801 | 319 | 13934 | 0.288401254 | 2.24E-14 | 7.33E-13 |
| ko04514 | Cell adhesion molecules (CAMs) | 103 | 1801 | 384 | 13934 | 0.268229167 | 1.09E-13 | 3.24E-12 |
| ko05150 | Staphylococcus aureus infection | 67 | 1801 | 207 | 13934 | 0.323671498 | 2.26E-13 | 6.16E-12 |
| ko04660 | T cell receptor signaling pathway | 60 | 1801 | 176 | 13934 | 0.340909091 | 3.23E-13 | 8.12E-12 |
| ko04060 | Cytokine-cytokine receptor interaction | 103 | 1801 | 393 | 13934 | 0.262086514 | 5.31E-13 | 1.24E-11 |
| ko04659 | Th17 cell differentiation | 74 | 1801 | 245 | 13934 | 0.302040816 | 6.57E-13 | 1.35E-11 |
| ko05332 | Graft-versus-host disease | 68 | 1801 | 216 | 13934 | 0.314814815 | 6.61E-13 | 1.35E-11 |
| ko04940 | Type I diabetes mellitus | 72 | 1801 | 236 | 13934 | 0.305084746 | 7.83E-13 | 1.51E-11 |
| ko05321 | Inflammatory bowel disease (IBD) | 59 | 1801 | 177 | 13934 | 0.333333333 | 1.52E-12 | 2.75E-11 |
| ko05142 | Chagas disease (American trypanosomiasis) | 58 | 1801 | 173 | 13934 | 0.335260116 | 1.78E-12 | 3.07E-11 |
| ko05322 | Systemic lupus erythematosus | 80 | 1801 | 287 | 13934 | 0.278745645 | 7.87E-12 | 1.29E-10 |
